# Comparative analysis of midgut bacterial communities in Chikungunya virus-infected and non-infected *Aedes aegypti* Thai laboratory strain mosquitoes

**DOI:** 10.1038/s41598-024-61027-0

**Published:** 2024-05-11

**Authors:** Padet Siriyasatien, Proawpilart Intayot, Suwalak Chitcharoen, Nataya Sutthanont, Rungfar Boonserm, Rinnara Ampol, Jonas Schmidt-Chanasit, Atchara Phumee

**Affiliations:** 1 Center of Excellence in Vector Biology and Vector Borne Diseases, Department of Parasitology, Faculty of Medicine, Chulalongkorn University, Bangkok, Thailand; 2 Pharmaceutical Ingredient and Medical Device Research Division, Research Development and Innovation Department, The Government Pharmaceutical Organization, Bangkok, Thailand; 3 Department of Microbiology, Faculty of Medicine, Khon Kaen University, Khon Kaen, Thailand; 4 Department of Medical Entomology, Faculty of Tropical Medicine, Mahidol University, Bangkok, Thailand; 5 Bernhard-Nocht-Institute for Tropical Medicine, Hamburg, Germany; 6 Faculty of Mathematics, Informatics and Natural Sciences, University of Hamburg, Hamburg, Germany; 7 Department of Medical Technology, School of Allied Health Sciences, Walailak University, Nakhon Si Thammarat, Thailand; 8 Excellent Center for Dengue and Community Public Health (EC for DACH), Walailak University, Nakhon Si Thammarat, Thailand

**Keywords:** Entomology, Infectious diseases

## Abstract

Chikungunya virus (CHIKV) poses a significant global health threat, re-emerging as a mosquito-transmitted pathogen that caused high fever, rash, and severe arthralgia. In Thailand, a notable CHIKV outbreak in 2019–2020 affected approximately 20,000 cases across 60 provinces, underscoring the need for effective mosquito control protocols. Previous studies have highlighted the role of midgut bacteria in the interaction between mosquito vectors and pathogen infections, demonstrating their ability to protect the insect from invading pathogens. However, research on the midgut bacteria of *Aedes* (*Ae.*) *aegypti*, the primary vector for CHIKV in Thailand remains limited. This study aims to characterize the bacterial communities in laboratory strains of *Ae. aegypti,* both infected and non-infected with CHIKV. Female mosquitoes from a laboratory strain of *Ae. aegypti* were exposed to a CHIKV-infected blood meal through membrane feeding, while the control group received a non-infected blood meal. At 7 days post-infection (dpi), mosquito midguts were dissected for 16S rRNA gene sequencing to identify midgut bacteria, and CHIKV presence was confirmed by *E1*-nested RT-PCR using mosquito carcasses. The study aimed to compare the bacterial communities between CHIKV-infected and non-infected groups. The analysis included 12 midgut bacterial samples, divided into three groups: CHIKV-infected (exposed and infected), non-infected (exposed but not infected), and non-exposed (negative control). Alpha diversity indices and Bray–Curtis dissimilarity matrix revealed significant differences in bacterial profiles among the three groups. The infected group exhibited an increased abundance of bacteria genus *Gluconobacter*, while *Asaia* was prevalent in both non-infected and negative control groups. *Chryseobacterium* was prominent in the negative control group. These findings highlight potential alterations in the distribution and abundance of gut microbiomes in response to CHIKV infection status. This study provides valuable insights into the dynamic relationship between midgut bacteria and CHIKV, underscoring the potential for alterations in bacterial composition depending on infection status. Understanding the relationships between mosquitoes and their microbiota holds promise for developing new methods and tools to enhance existing strategies for disease prevention and control. This research advances our understanding of the circulating bacterial composition, opening possibilities for new approaches in combating mosquito-borne diseases.

## Introduction

Chikungunya virus (CHIKV), an alphavirus in the *Togaviridae* family, is transmitted by *Aedes* (*Ae*.) mosquitoes, primarily *Ae. aegypti* and *Ae. albopictus*^1^. In Asia, *Ae. aegypti* is the primary vector of CHIKV in epidemics^2^. The virus was first isolated in 1953 from febrile patients and mosquitoes from the Newala district of Tanzania^3^. CHIKV comprises three genotypes: western African, east-central-south African (ECSA), and Asian genotype^4,5^. CHIKV infection has emerged as a serious public health concern worldwide, leading to outbreaks in large tropical areas across Africa, Asia, Europe, and the Americas^6,7^. In Thailand, between 2017 and 2022, the Thai Ministry of Public Health annually reported CHIKV cases, with over 30,000 confirmed cases recorded (10 cases in 2017, 3580 cases in 2018, 13,121 cases in 2019, 11,331 cases in 2020, 671 cases in 2021, and 1311 cases in 2022)^8^. The number of reported cases began surging in 2018, and by 2019–2020, the outbreak had spread to over 60 provinces across the country. Additionally, reports indicated that CHIKV RNA was detected in 3.28% of the female and 0.85% of the male *Ae. aegypti* mosquito samples collected during the 2019–2020 outbreak^9^. These CHIKV RNA positive samples suggested the presence of vertical transmission in the field population of *Ae. aegypti*. Moreover, analysis of the genetic diversity of CHIKV in field-caught *Ae. aegypti* mosquitoes revealed notable mutations. The E1: A226V mutation was found in females, and the E1: K211E mutation in both females and males^9,10^. At present, there are no commercially available vaccines or specific drugs for the treatment of Chikungunya fever. The current treatment primarily focuses on relieving symptoms^11^. As CHIKV continues to increase prevalence, geographical distribution and severity, available control options remain limited^12^. Moreover, the unpredictable re-emergence of CHIKV outbreaks in Thailand emphasizes the importance of relying on vector mosquito control measures for effective disease control.

Chemical control was once the primary strategy for controlling mosquito-borne diseases. However, concerns about the environment and human health impact of available compounds, coupled with the development of insecticide resistance in mosquitoes, have constrained the effectiveness of this approach. Nowadays, there has been growing recognition of the potential role of gut microbiota as symbiotic bacteria capable of influencing the control of mosquito-borne diseases and reducing the transmission of pathogens in mosquitoes^13,14^. Therefore, our research explores novel methods for CHIKV control, focusing on vector-associated bacteria. Several studies have suggested that microbial symbionts in mosquitoes play an important role in host biology and offer potential avenues for mosquito control^15–19^. In 2018, Muturi and others showed that the diversity in microbial composition and mosquito species from different distinct geographic areas could have important implications for vector competence and transmission dynamics of mosquito-borne pathogens. Various bacteria are commonly found in the mosquito gut, germline tissues, Malpighian tubules, and salivary glands^15,16^. Sharma et al. discovered a more diversified microbiota in the salivary gland compared to the gut of *Anopheles culicifacies,* with 11% similarity between the symbiotic bacterial communities of the midgut and salivary gland, both involved in food digestion^17^. Common taxa, including *Asaia, Enterobacter, Pseudomonas*, and *Serratia,* are shared between *Aedes* and *Anopheles* vectors^18,19^. Many studies identified Actinobacteriota such as *Streptomyces, Microbacterium*, and *Micrococcus*; Firmicutes such as *Bacillus*; and Proteobacteria such as *Asaia, Chromobacterium, Enterobacter, Pantoea*, *Pseudomonas*, and *Serratia* in *Ae. aegypti* and *Ae. albopictus*^20,21^. In Thailand, Thongsripong et al. found that diversity within the field-collected *Ae. aegypti*, *Ae. albopictus*, and *Culex. quinquefasciatus* mosquito microbiota community composition depends on factors like habitat condition and mosquito species^22^. In 2018, Tiawsirisup and others suggested that there were differences in the bacterial genera found in the midgut of laboratory-reared and field-collected female *Ae. aegypti*, potentially due to differences in environmental conditions^23^. Microbiome evidence revealed an impact on arboviruses, for instance, CHIKV infection increases the abundance of bacteria in the family *Enterobacteriaceae* and reduces *Wolbachia* and *Blattabacterium*^24^. Moreira et al. argued that *Wolbachia* infection in *Ae. aegypti* reduced vector competence and replication of dengue virus (DENV), CHIKV, *Plasmodium gallinaceum* and filarial nematodes^25^. Wu et al. revealed that *Serratia marcescens* enhances the susceptibility of field *Ae. aegypti* mosquitoes to Dengue serotype 2 virus (DENV-2)^26^. These results indicated that variations in the midgut microbiome could influence a mosquito’s vector competence for specific pathogens.

However, information regarding gut microbiota in mosquitoes and interactions between bacterial and viral pathogens in mosquitoes from Thailand is limited. Therefore, this study focuses on comparing the interesting bacterial communities of adult *Ae. aegypti* mosquitoes with CHIKV-infected group (exposed and infected), non-infected group (exposed but not infected), and non-exposed group (negative control). We use MiSeq sequencing of the 16S rRNA gene to identify bacterial microbes in *Ae. aegypti* with various CHIKV infection status, including non-infected mosquitoes. The microbial community richness, diversity, and composition are then compared between CHIKV-infected and non-infected to determine their association with CHIKV infection in *Ae. aegypti*. The findings of this study aim to enhance our understanding of CHIKV infected and non-infected in *Ae. aegypti* mosquito-microbe interactions. Identifying key microbial taxa could potentially lead to developing novel and efficient strategies for controlling chikungunya fever and other mosquito-borne viral diseases.

## Results

At 7 days post-infection (dpi), approximately 50 mosquitoes exhibited distended abdomens with no visible signs of undigested blood. However, due to challenges encountered during dissection, only 22 of the 50 mosquito midguts were successfully collected for further analysis. All 22 mosquito carcasses yielded 13 positive (infected group) and 9 negative (non-infected group) CHIKV RNA samples (Supplementary Fig. S1). In our comparison of bacterial microbes across various CHIKV infection statuses, we included a total of 12 samples of midgut bacteria. This set consisted of 6 positive CHIKV RT-PCR samples (infected group), 6 negative CHIKV RT-PCR samples (non-infected group), and 2 samples of negative control group (Table 1).

**Table 1:** Sample information.

Group	Results of RT-PCR	Code
CHIKV-infected (exposed and infected)	Positive	MP1
	Positive	MP2
	Positive	MP3
	Positive	MP4
	Positive	MP5
	Positive	MP6
Non-infected (exposed but not infected)	Negative	MN1
	Negative	MN2
	Negative	MN3
	Negative	MN4
	Negative	MN5
	Negative	MN6
Non-exposed (negative control)	Negative	uninfect01
	Negative	uninfect02

**Figure 1:**
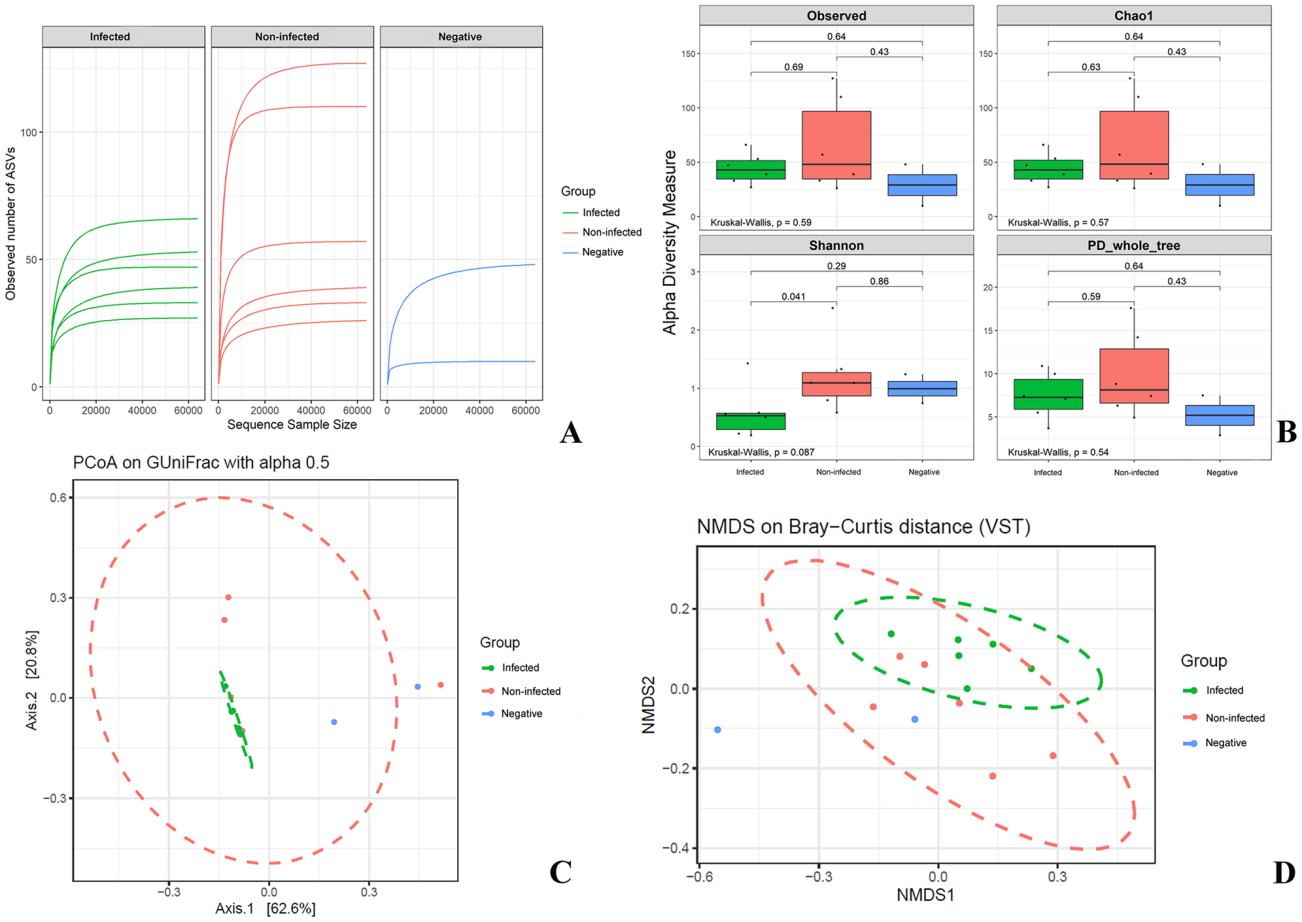
Representations of the plateau curve in Rarefaction curves (**A**), Boxplot representations of Alpha-diversity indices (**B**), Beta diversity analyses included GUniFrac with an alpha value of 0.5 distance (**C**) and NMDS analysis based on Bray–Curtis dissimilarity (**D**), with the infected group shown in green, the non-infected group in pink, and the negative control group in blue. All figures were modified from free software under public domain or a free license.

The estimated saturation of microbial richness in all samples was reached at 63,898 sequencing depths, as indicated by the rarefaction curves. A plateau curve in rarefaction was observed at a sequencing depth of approximately 20,000, suggesting that the true bacterial compositions of the gut microbiome were sufficiently estimated for all sample groups. The MN2 and MN3 samples from the non-infected group exhibited the highest number of observed ASVs compared to the CHIKV-infected and negative control groups. In contrast, the negative control group had the lowest number of observed ASVs. These findings suggest that different conditions can affect the abundance of gut microbiota (Fig. 1A). For Alpha diversity analyses, the high-quality reads of the 16S rRNA after processing totaled 1,095,649 reads. The observed abundance of ASVs, bacterial abundance (Chao1 index), diversity (Shannon index), and PD whole tree showed no significant difference between the sample groups (Kruskal–Wallis test; *p* = 0.59, 0.57, 0.087 and 0.54, respectively). However, the Shannon index showed a statistically significant difference between the infected and non-infected groups (*p* = 0.041), indicating that the infected group had the lowest bacterial diversity (Fig. 1B). For Beta diversity analyses, the weighted UniFrac PCoA, GUniFrac, and NMDS based on Bray–Curtis distance suggested that microbiota communities of the infected and negative control groups were clearly distinct (PERMANOVA test; p = 0.03, 0.024, and 0.006, respectively). Additionally, distance metric analysis of weighted UniFrac and GUniFrac with alpha 0.05 showed that the gut microbial structures of the negative control group were significantly different from those of both the infected and non-infected groups (Wilcoxon test; *p* < 0.01) (Fig. 1C,D). This suggests distinct gut microbial communities in the infected and negative control groups.

**Figure 2:**
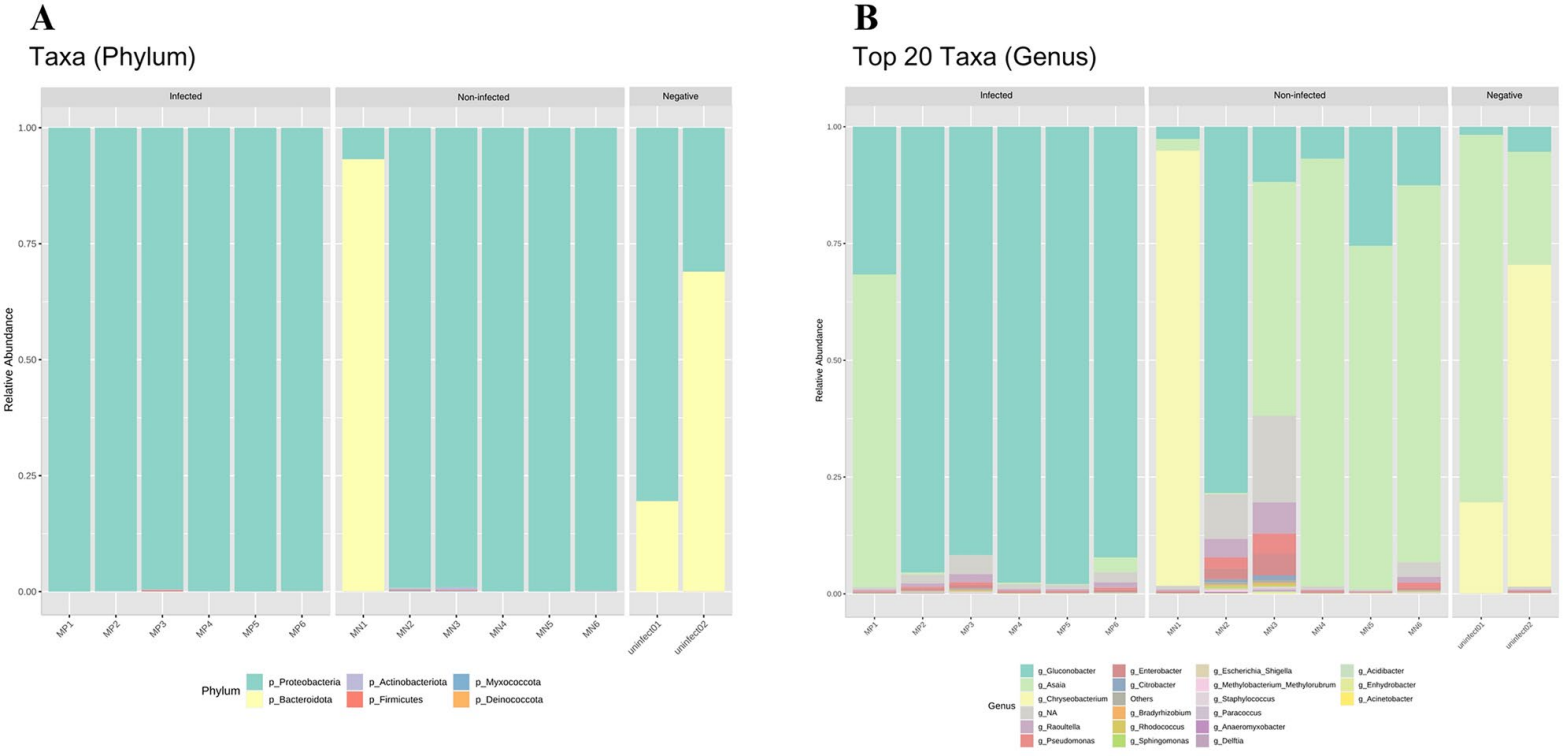
Barplots showing the taxonomic profiles at the phylum (**A**) and genus (**B**) level of the top 20 most abundant groups in terms of relative abundance of infected, non-infected, and negative control groups by high throughput 16S ribosomal RNA gene sequencing. All figures were modified from free software under public domain or a free license.

Eleven different bacterial phyla were identified in the mosquito midgut samples: Actinobacteriota, Aquificota, Bacteroidota, Chloroflexi, Cyanobacteria, Deinococcota, Desulfobacterota, Firmicutes, Myxococcota, Proteobacteria, and Verrucomicrobiota. Proteobacteria was the most highly prevalent phylum (average 0.86 ± 0.07), followed by Bacteriodota and Actinobacteriota, respectively (Fig. 2A). Proteobacteria dominated in the infected group (0.99 ± 0.001) and the non-infected group (0.83 ± 0.15). In the negative control group, the abundance of Proteobacteria decreased (0.55 ± 0.24), while the abundance of Bacteriodota increased (0.44 ± 0.24) compared to the other groups. Bacteria in the class of Bacteroidia increased in the negative control group, while Proteobacteria was relatively high in the infected and non-infected groups. Overall, 121 genera were detected among samples. The relative abundance of *Gluconobacter* bacteria was significantly increased in the infected group compared to the other groups (*p* < 0.05) (Fig. 2B). This finding suggests that the gut microbiome of mosquito varied according to the conditions.

**Figure 3:**
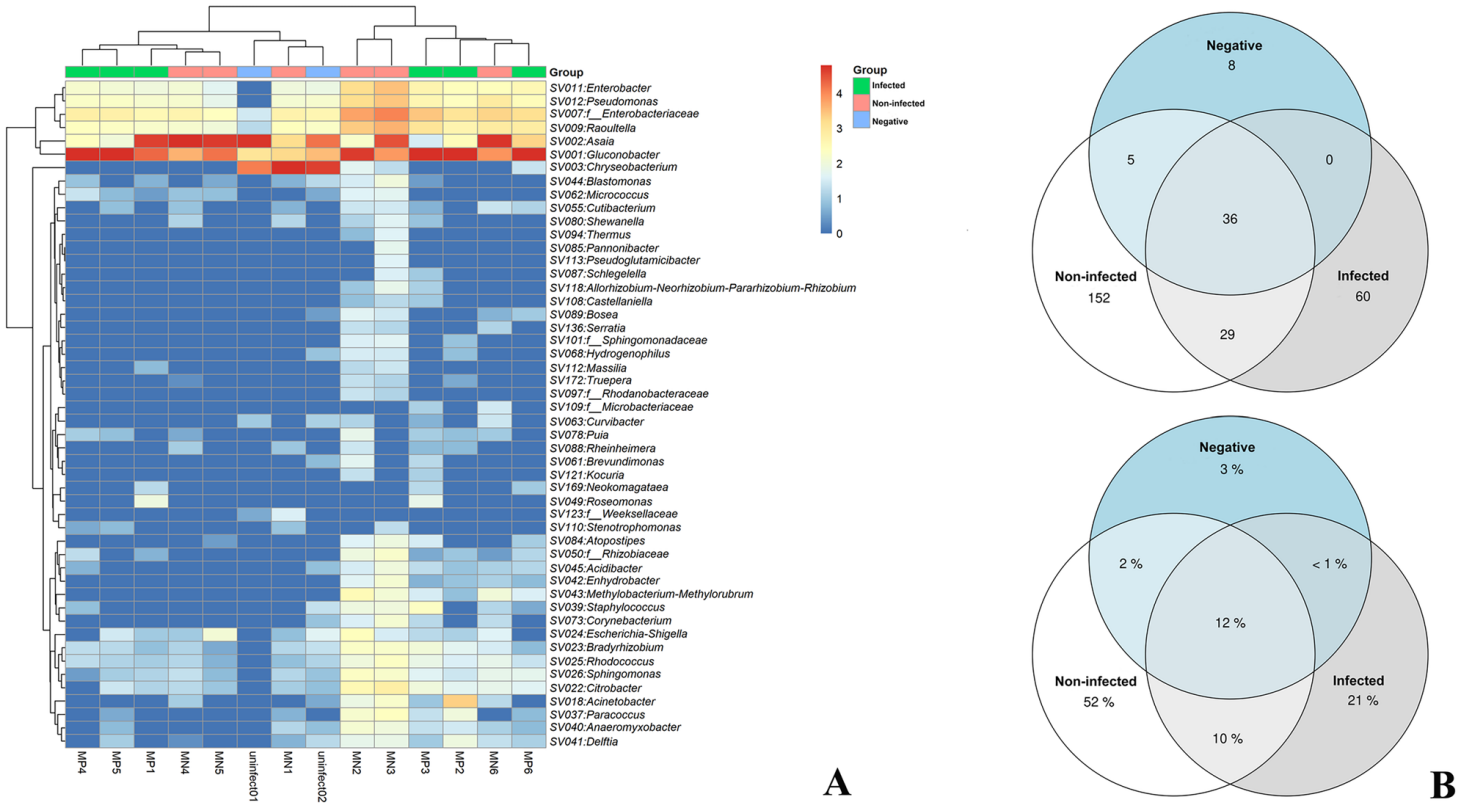
Heatmap of the log relative abundance of top genera (**A**) and Venn diagram of shared 16S rRNA OTUs (**B**) from the infected, non-infected, and negative control groups. All figures were modified from free software under public domain or a free license.

A heatmap of the dominant genera showed the shifts in microbial compositions. The genera *Enterobacter*, *Pseudomonas*, *Enterobacteriaceae*, *Raoultella*, *Asaia*, and *Gluconobacter* were highly detected in all samples. However, *Gluconobacter* was significantly enriched in the infected group. Interestingly, *Asaia* was found in non-infected and negative control groups, while *Chryseobacterium* was highly present in the negative control group. This result suggests that the distribution and abundance of the gut microbiome in mosquitoes could vary according to different conditions (Fig. 3A). A Venn diagram analysis of the core, shared, and individual microbiomes among groups showed that 36 ASVs (12%) were common to all groups, while 152, 60, and 8 ASVs were unique to the non-infected, infected, and negative control groups, respectively. The non-infected and negative control groups shared the lowest number of ASVs (41 ASVs or 14%). The infected group shared 65 ASVs with the non-infected group and 36 ASVs with the negative control group. Overall, 36 ASVs were considered the core microbiota of the mosquito gut microbiome (Fig. 3B).

**Figure 4:**
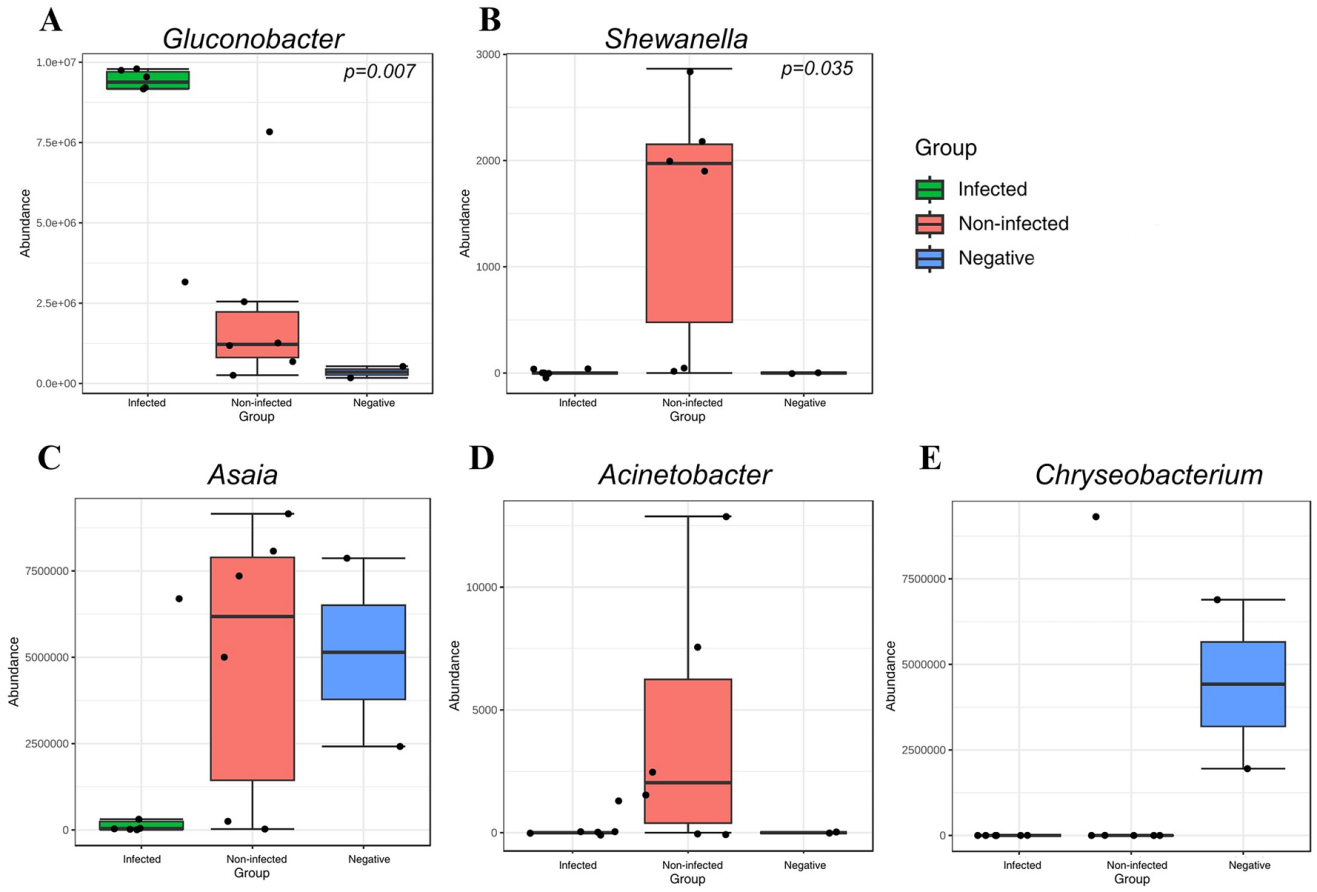
Genus level distribution and linear discriminant analysis (LDA) effect size (LEfSe) analysis of *Gluconobacter* (**A**), *Shewanella* (**B**), *Asaia* (**C**), *Acinetobacter* (**D**), and *Chryseobacterium* (**E**) revealed differences in the gut microbiota among the infected, non-infected, and negative control groups. All figures were modified from free software under public domain or a free license.

Linear discriminant analysis effect size (LEfSe) was used to identify bacterial taxa that differed significantly between groups. Bacterial taxa with LDA scores greater than 2 were considered significant (*p* < 0.05). The genus *Gluconobacter* emerged as the core gut microbiota in infected group (*p* < 0.05), indicating that this bacterium played a role in CHIKV infection conditions compared to both the non-infected and the negative control groups (Fig. 4A). The abundance of *Shewanella* (Fig. 4B), *Asaia* (Fig. 4C), and *Acinetobacter* (Fig. 4D) was highly enriched in the gut microbiome of the non-infected group. Moreover, we found that gut microbiome of the negative control group differed from the infected group, with an increased abundance of *Chryseobacterium* (Fig. 4E). These findings suggest the distribution and abundance of gut microbiome in each group could be changed according to the CHIKV-infection status.

## Discussion

CHIKV infection in humans may cause fever, joint pain, and rash. CHIKV is transmitted to humans through bites from infected mosquitoes^27^. *Ae. aegypti* is a known vector of CHIKV; its abundance in a region is a major factor in the transmission of the virus^28^. Efforts to control this mosquito species can help reduce the spread of CHIKV and other mosquito-borne diseases. New strategies propose manipulating mosquito hosts and their associated bacterial communities^29,30^. This study investigated the bacterial communities in the midguts of *Ae. aegypti* infected with CHIKV including infected group (exposed and infected), non-infected group (exposed but not infected), and negative control group using 16S rDNA gene sequencing. The results showed that the core gut microbiota in the infected group was identified as *Gluconobacter*, an acetic acid bacterium in the Alpha-proteobacteria class. Conversely, bacterial genera *Asaia* (Alpha-proteobacteria), *Shewanella* (Gamma-proteobacteria), and *Acinetobacter* (Gamma-proteobacteria) were highly enriched in the gut microbiome of the non-infected group. *Chryseobacterium* (Flavobacteriia) was found in the negative control group. These findings suggest that the distribution and abundance of gut microbiomes in each group can be influenced by the CHIKV-infection status. A previous study by Muturi et al. revealed similarities in bacterial communities among *Aedes*, *Anopheles*, and *Culex* in the USA, including the presence of the genera *Gluconobacter*, *Propionibacterium*, and *Staphylococcus*^31^. *Gluconobacter*, a group of acetic acid bacteria, has been shown to be adaptable to a wide range of environments rich in sugars and ethanol^32^. Remarkably, several reports have highlighted the presence of *Gluconobacter* in insects, particularly mosquitoes, which primarily rely on sugar-based diets^33,34^. Notably, our study identified *Gluconobacter* in all groups, displaying the highest abundance in the infected group, followed by the non-infected and negative control groups (*p* = 0.007). Our findings suggest that *Gluconobacter* may potentially increase the susceptibility of *Ae. aegypti* to CHIKV. To the best of our knowledge, this is the first study to report the identification of *Gluconobacter* in CHIKV-infected *Ae. aegypti*, shedding light on a previously unexplored aspect of the interaction between *Gluconobacter* and CHIKV infection. This contributes to our understanding of mosquito vector competence in the context of this viral pathogen. Further investigations are warranted to delve deeper into this relationship and better understand its implications. As for the genus *Asaia*, a member of the *Acetobacteraceae* family, it is well-documented for establishing symbiotic associations with mosquitoes^35^. These interactions between *Asaia* and mosquitoes have been a subject of scientific interest due to their potential significance in mosquito biology, ecology, and vector competence^15,33,34^. In our study, *Asaia* was predominantly observed in the non-infected group, with slightly presence in the infected group. These findings suggest the possibility that *Asaia* might play a role in inhibiting CHIKV in *Ae. aegypti*. The bacterium *Asaia* is considered a highly promising candidate for arboviral control in *Aedes* mosquitoes, given its well-documented adaptability to colonize both laboratory and field mosquitoes^30,36–39^. Furthermore, *Asaia* has been employed in paratransgenesis for malaria control, revealing its ability to hinder larval development in *Anopheles* spp.^36^. Additionally, these findings underscore the versatile potential of *Asaia* for vector-borne disease control in different mosquito species. However, it is important to note that the available research on the capacity of *Asaia* to reduce CHIKV infection remains limited. Zouache et al. suggested an increase in the prevalence of bacteria belonging to the *Enterobacteriaceae* family in response to CHIKV infection, while well-documented insect endosymbionts such as *Wolbachia* and *Blattabacterium* decreased in *Ae. albopictus*^24^. Moreover, the isolation of *S. odorifera* has been demonstrated to enhance the replication of both DENV and CHIKV in *Ae. aegypti*^40,41^. This increased susceptibility of female *Ae. aegypti* mosquitoes to CHIKV may result from the suppression of the immune system, occurring due to the interaction between the P40 protein from *S. odorifera* and the porin protein on the gut membrane of *Ae. aegypti*^40^. However, the bacterial community undergoes dynamic changes throughout the life cycle of mosquitoes, with composition variations based on factors such as mosquito gender, developmental stage, and ecological conditions^42^. Therefore, these variations in microbiota composition may help elucidate the vector competence commonly observed across mosquito populations^43^. Additionally, microbiota can influence mosquito development^19^, nutrient acquisition^44^, blood digestion^45^, and the synthesis of the peritrophic matrix^46^. This study provides fundamental data on the microbiota associated with both CHIKV-infected and non-infected *Ae. aegypti* mosquitoes of the Thai laboratory strain*.* For future investigations, we plan to conduct extensive surveys and more precise studies of CHIKV-infected and non-infected *Ae. aegypti* collected from field sites in Thailand. This data is crucial for understanding the geographical, habitat, and ecological interactions between the microbiota and CHIKV in *Ae. aegypti*. In addition, we will perform a culture-dependent approach to gain insights into the bacterial diversity within the midgut of *Ae. aegypti* and its interaction with CHIKV. Despite facing difficulties in culturing gut bacteria and limitations in sample size, our research successfully lays valuable groundwork for future investigations. These studies can build upon our work by exploring alternative methods to delve into the intricacies of mosquito gut microbiome and its potential role in disease transmission.

## Conclusions

Viruses transmitted by mosquitoes, such as CHIKV, pose an ongoing threat to human health. In the absence of vaccines or specific treatments, controlling mosquitoes or reducing their virus-transmitting capacity remains the key strategy for preventing mosquito-borne viral diseases. Although understanding of mosquito microbiota’s influence on CHIKV transmission has been primarily based on association studies, our research suggests that *Gluconobacter* might increase the susceptibility of *Ae. aegypti* to CHIKV infection. Conversely, *Asaia* could play a role in inhibiting CHIKV within *Ae. aegypti*. These findings illuminate the complex interplay between mosquito-associated bacteria and CHIKV transmission, contributing to a more profound understanding of vector competence.

## Methods

### Mosquitoes

Laboratory colonies of *Ae. aegypti* mosquitoes were maintained under standard conditions as follows: 28 ± 2 °C, 65–85% relative humidity, and a 12/12-h light/dark cycle. These mosquitoes were initially collected as eggs from Nonthaburi Province in Central Thailand in 2007. The populations of *Ae. aegypti* have been reared for 331 generations. Adult mosquitoes were provided with a mixture of 5% sucrose and 5% vitamin B complex (w/v)^47,48^ for ad libitum consumption, while larvae were raised in plastic trays and fed with minced commercial mouse food until reaching the pupal stage.

### Virus strain

The CHIKV was isolated from female *Ae. aegypti* mosquitoes that were collected during an epidemic in Bangkok, Thailand^10^. Specifically, the virus was maintained in *Ae. albopictus* C6/36 insect cells (ATCC CRL-1660), which were cultured in Minimum Essential Medium (MEM) supplemented with 10% heat-inactivated Fetal Bovine Serum (FBS) (Gibco, USA). The cultivation process was performed at a temperature of 28 °C with 5% CO_2_ for three more passages. The CHIKV strain was classified as belonging to the Indian Ocean clade, which falls under the East-Central South African (ECSA) genotype. Subsequently, viral stocks were cultivated in C6/36 cells and preserved at a temperature of − 80 °C for future use.

### Mosquito infection with CHIKV

**Figure 5:**
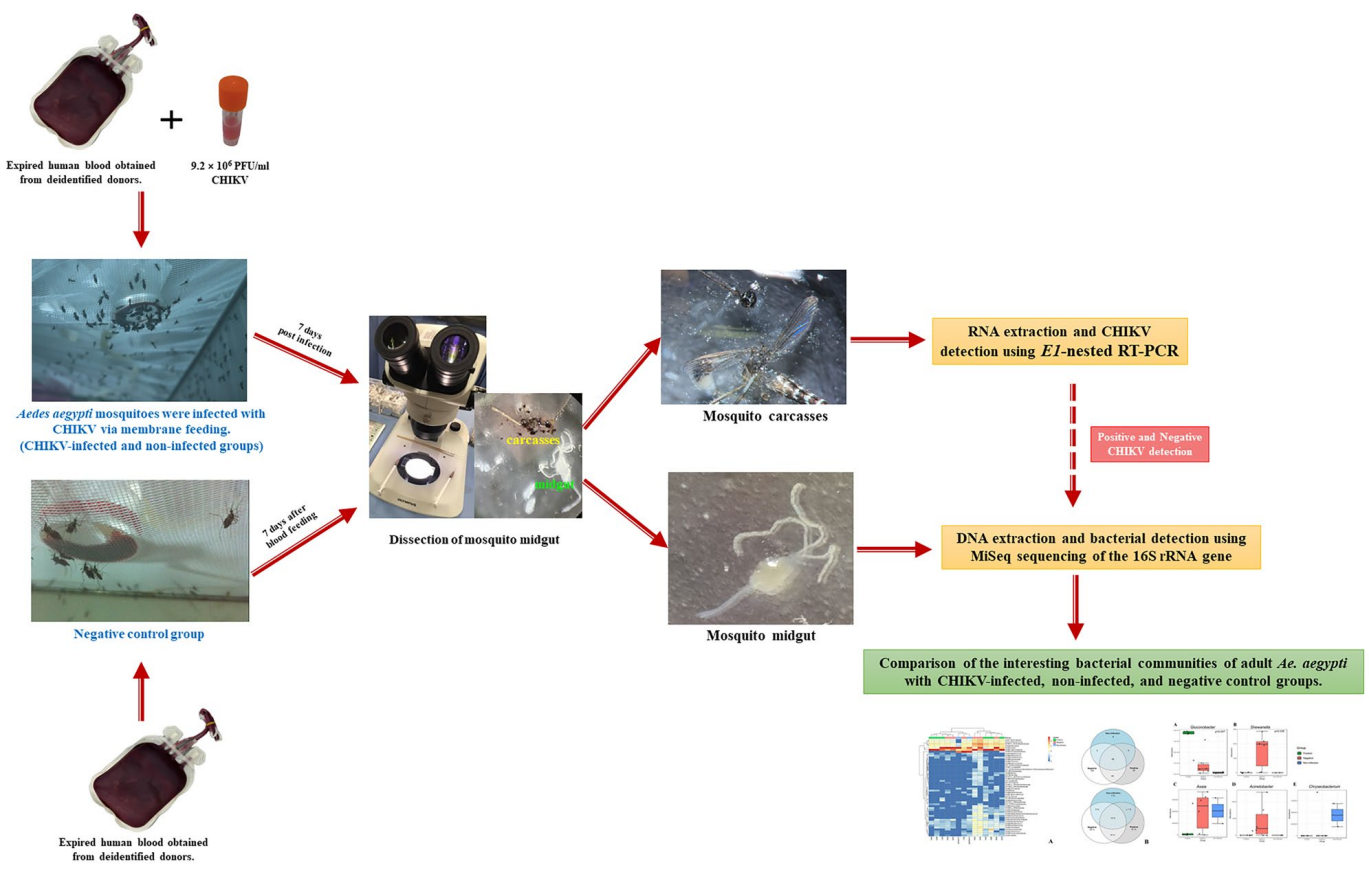
Conducting laboratory experiments and processing systems infected with CHIKV within mosquitoes. All images were captured and edited by Atchara Phumee and the co-authors.

The female mosquitoes were fed using an artificial membrane feeding. Artificial blood feeding was performed according to the technique described by Dias et al.^49^ using a circulating water bath (Thermo-Scientific, USA) set to maintain warm water (approximately 37 °C). The water circulated through thin hoses connected to jacketed glass cones with a small opening at the top and a large concave base. The base aperture was occluded by a stretched Parafilm-M, mimicking real animal skin. We opted for an artificial membrane feeding system to allow for controlled blood meal composition and minimize contamination risks. Prior to performing the oral infection, the viral titer of the virus stock was determined to be 9.2 × 10^6^ PFU/ml. Five-day-old female *Ae. aegypti* were starved for 24 h before being fed with expired human blood obtained from deidentified donors^50^, which tested negative for CHIKV RNA. The blood was sourced from the National Blood Center, Thai Red Cross Society, Bangkok, Thailand. The starved females were fed via artificial blood feeding at 37 °C under complete darkness. The feeding duration was set for 60 min. Non-engorged females were removed, while engorged females were transferred to a new container and provided with a diet consisting of 5% sucrose and 5% vitamin B complex (w/v). At 7 days post infection (dpi), individual mosquitoes were anesthetized and dissected in a drop of 1X PBS on a glass slide under a stereomicroscope (Olympus, Japan). The midgut from each female mosquito was collected to detect the presence of bacterial microbes, and mosquito carcasses were also analyzed for the purpose of detecting CHIKV (Fig. 5).

### CHIKV RNA detection by *E1*-nested RT-PCR

The carcasses of individual mosquitoes were mixed with 400 µl of lysis buffer and processed for viral RNA extraction using the Invisorb Spin Virus RNA Mini viral RNA extraction kit (STRATEC Molecular GmbH, Germany) following the manufacturer’s instructions. The RNA was subjected to amplification and tested for CHIKV detection using nested RT-PCR. The first amplification was performed using two outer primer pairs that targeted the *E1* gene of CHIKV [E1-10145 F: 5′-ACAAAACCGTCATCCCGTCTC-3′ genome position 10,145–10,165 and E1-11158R: 5′-TGACTATGTGGTCCTTCGGAGG-3′ genome position 11,137–11,158]^51^. Subsequently, for the second amplification, newly designed inner primers based on *E1* gene sequences were employed, with the forward primer as 5′-GCGCCTACTGCTTCTGCGA-3′ and the reverse primer as 5′-CTTCATCGCTC TTACCGGGT-3′. The first round of PCR reactions was conducted in a final volume of 25 µl using the Superscript III one-step RT-PCR kit (Invitrogen, USA). The PCR cycling conditions included an initial incubation at 50 °C for 30 min, denaturation at 95 °C for 15 min, followed by 40 cycles of 95 °C for 1 min, 64 °C for 1 min, 72 °C for 1 min, and a final extension at 72 °C for 10 min. Two microliters of the first amplification product were then further amplified using the inner primer pairs in a final volume of 25 µl. The reaction mixture underwent amplification with the following parameters: 95 °C for 3 min, followed by 40 cycles of 95 °C for 30 s, 62 °C for 30 s, 72 °C for 1 min, and a final step at 72 °C for 7 min. All tested negative for non-template control (NTC) using double-distilled H_2_O (ddH_2_O) and negative control (uninfected *Ae. aegypti* RNA). The amplified products were subsequently analyzed using a 1.5% agarose gel, stained with ethidium bromide, and visualized under ultraviolet light using Quantity One Quantification Analysis Software version 4.5.2 (Gel DocEQ System; Bio-Rad, USA). The identity of CHIKV RNA was confirmed by determining the size of the amplicon, which measured approximately 539 base pairs (bp) in length.

### 16S rRNA library sequencing

Genomic DNA was extracted from individual specimens of midgut (6 samples of exposed and infected mosquito, 6 samples of exposed but not infected mosquito and 2 samples of non-exposure mosquito) using the Invisorb Spin Tissue Mini Kit (STRASTEC Molecular GmbH, Germany) as per the manufacturer’s instructions. The non-template control using ddH_2_O were used as negative control. The prokaryotic 16S rRNA gene at V3V4 region was performed using the Qiagen QIAseq 16S/ITS Region panel (Qiagen, Germany). 16S rRNA amplicons were labeled with different sequencing adaptors using QIAseq 16S/ITS Region Panel Sample Index PCR Reaction (Qiagen, Germany). The quality and quantity of the resulting DNA libraries, approximately 630 bp in size, were evaluated using QIAxcel Advanced (Qiagen, Germany) and DeNovix QFX Fluorometer, respectively. Finally, 16S rRNA libraries were sequenced using an illumina Miseq600 platform (Illumina, USA).

### Bioinformatics analyses

The raw sequences were first grouped based on their unique 5′ barcode sequences. These barcode-sorted sequences were then processed using the DADA2 v1.16.0 pipeline (https://benjjneb.github.io/dada2/). This pipeline is instrumental in identifying and quantifying unique amplicon sequence variants (ASVs), renowned for its efficacy in unraveling microbial diversity and community structures^52^. Microbial taxonomy was assigned using Silva version 138 as the reference database^53^. Alpha diversity metrics, including Chao1 richness, Shannon, and PD whole tree, were evaluated utilizing the DADA2 software. For beta diversity analysis, non-metric multidimensional scaling (NMDS) based on Bray–Curtis dissimilarity and principal coordinate analysis (PCoA) were conducted using Phyloseq data. Linear discriminant analysis effect size (LEfSe) and cladogram plots were generated to identify bacterial biomarkers. A Venn diagram was used to illustrate the core microbiome constituents shared across all samples. In delving into bacterial correlated evolution, a phylogenetic tree was exhaustively constructed.

### Statistical analysis and data analysis

The pairwise comparison of alpha diversity indices, including observed ASVs, Chao1 richness, Shannon diversity, and PD whole tree diversity, were performed using the Kruskal–Wallis test (*p* < 0.05). To assess the statistical significance of beta diversity differences among groups, a Permutational Multivariate Analysis of Variance (PERMANOVA) was conducted using a significance level of *p* < 0.05. Additionally, the Kruskal–Wallis sum-rank test (*p* < 0.05) was used within the LEfSe analysis to identify bacterial biomarkers that significantly differentiated abundant taxa between sample groups.

### Ethics declarations

The study was approved by the animal research ethics committee of Chulalongkorn University and adhered to the Animal Care and Use Protocol (CU-ACUP). The Faculty of Medicine, Chulalongkorn University, Bangkok, Thailand (COA No. 021/2563) All experimental protocols requiring biosafety were approved by Institutional Biosafety Committees (IBC) of the Faculty of Medicine, Chulalongkorn University, Bangkok, Thailand (MDCU-IBC019/2020). The study does not involve human participants; therefore, a consent form was not required. This is because we utilized expired human blood from a blood bank, routinely discarded as biological waste. It is crucial to emphasize that the study received courtesy in the form of expired human blood from the National Blood Center, Thai Red Cross Society, Bangkok, Thailand.

### Supplementary Information

Supplementary Figure S1.

## Data Availability

The datasets used and/or analyzed during the current study are available from the corresponding author on reasonable request. All 16S rRNA gene sequences from this study have been deposited in NCBI’s SRA database under BioProject ID: PRJNA1043583 with accession number SRR2692512-SRR26912525.
