# Supplementary material for: Comparative analysis of midgut bacterial communities in Chikungunya virus-infected and non-infected Aedes aegypti Thai laboratory strain mosquitoes
Source: Sci Rep. 2024 May 11;14:10814. doi: 10.1038/s41598-024-61027-0 (PMC11088667; doi:10.1038/s41598-024-61027-0)
Supplement: Supplementary file 1 — Supplementary Figure S1. [file 41598_2024_61027_MOESM1_ESM.docx]

**
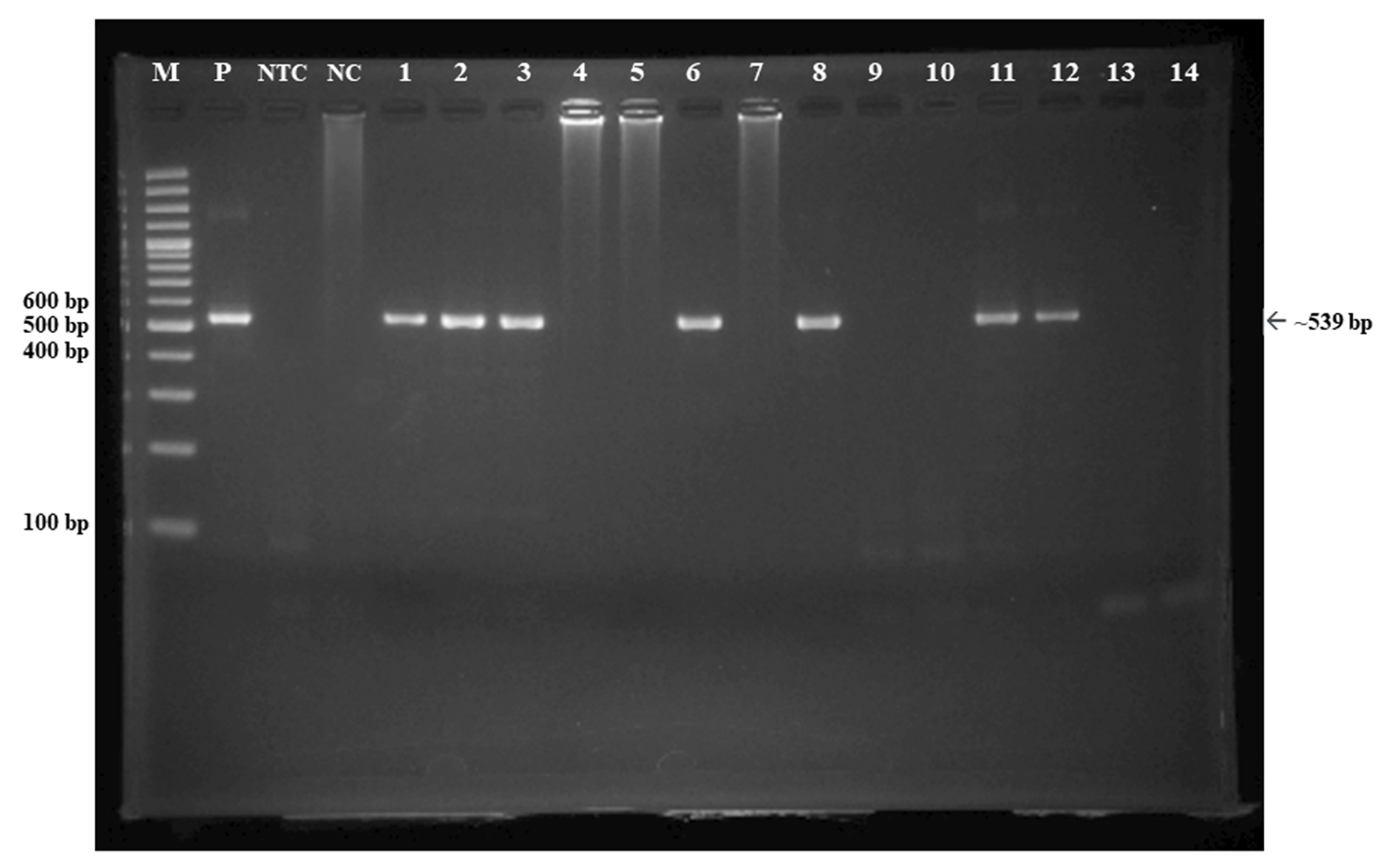
**

**Supplementary Figure S1** Agarose gel electrophoresis of PCR amplicons after amplification of *E1-*nested RT-PCR for CHIKV. Viral RNA extraction from carcasses of individual mosquitoes was used as template in *E1*-nested RT-PCR. PCR products were analyzed by electrophoresis through 1.5% agarose gels. Gel was stained with ethidium bromide and PCR products (approximately 539 bp) were visualized under UV light. Lane M: 100 bp DNA ladder, lane P: positive control, lane NTC: non-template control using ddH_2_O, lane NC: negative control (uninfected *Ae. aegypti* RNA), and lane 1-14: mosquito samples
